# The role of CARDPC in response to COVID-19 in primary care in China

**DOI:** 10.1038/s41533-020-00199-4

**Published:** 2020-09-18

**Authors:** Zihan Pan, Ting Yang, Chunhua Chi, Chen Wang

**Affiliations:** 1 Department of General Practice, Peking University First Hospital, Beijing, China; 2 Department of Pulmonary and Critical Care Medicine, Peking University Third Hospital, Beijing, China; 3 Department of Pulmonary and Critical Care Medicine, China-Japan Friendship Hospital, Beijing, China; 4 National Clinical Research Center for Respiratory Diseases, Beijing, China; 5 Institute of Respiratory Medicine, Beijing, China; 6 Chinese Academy of Medical Science, Beijing, China; 7 Chinese Academy of Medical Sciences & Peking Union Medical College, Beijing, China

**Keywords:** Health policy, Disease prevention

## Abstract

COVID-19 is wreaking havoc around the world, which is a serious challenge to all our health systems. China reacted quickly in the early stage of the pandemic, and accumulated a lot of experiences, especially in the prevention and control of COVID-19 at the primary care level. Here, we would like to share how the Chinese Alliance for Respiratory Diseases in Primary Care (CARDPC) played a role in the pandemic, hoping to provide guidance and hope for effective control of the outbreak worldwide, for future public health emergencies and for systematic management of chronic respiratory diseases in the community.

Since December of 2019, novel coronavirus disease 2019 (COVID-19) spread rapidly in China from Wuhan, the epicenter of the pandemic in China [http://www.nhc.gov.cn/xcs/yqtb/list_gzbd]. Four million medical staff working in primary medical facilities in China have participated in the response to COVID-19 in communities. The pandemic was a great challenge to China’s health system, which required all types of medical institutions, including primary care institutions, to join hands to meet the challenge. Currently, there is no “first contact in primary care” in China^1^; i.e. people are free to choose any medical institution or facility, but primary care plays an important role in health management. As an important organization for respiratory diseases at the primary care level, the Chinese Alliance for Respiratory Diseases in Primary Care (CARDPC) played a bridging role by connecting primary care medical facilities and tertiary care in this pandemic, and it carried out a series of effective measures to support primary care to deliver prevention and control of COVID-19 in communities.

## Introducing CARDPC

Respiratory diseases represent a heavy burden in China. The latest studies showed that there are 99 million patients with chronic obstructive pulmonary disease (COPD)^2^ and 43 million patients with asthma^3^, but fewer than 8,000 respiratory health physicians in the country. It is impossible for respiratory health physicians working in general hospitals or specific hospitals to manage such a large population with chronic respiratory diseases. CARDPC was founded in 2015 with the support of Chinese Thoracic Society (CTS) to solve the priority problems of management of respiratory diseases in primary care and to optimize the capability building of primary care physicians [https://www.cardpc.org/]. The roles and responsibilities of the alliance are to promote the standardized management of common respiratory diseases in primary care by providing systematic training, developing and disseminating guidelines applied in primary care, and exploring the referral mechanisms between primary care and higher level institutions.

**Fig. 1:**
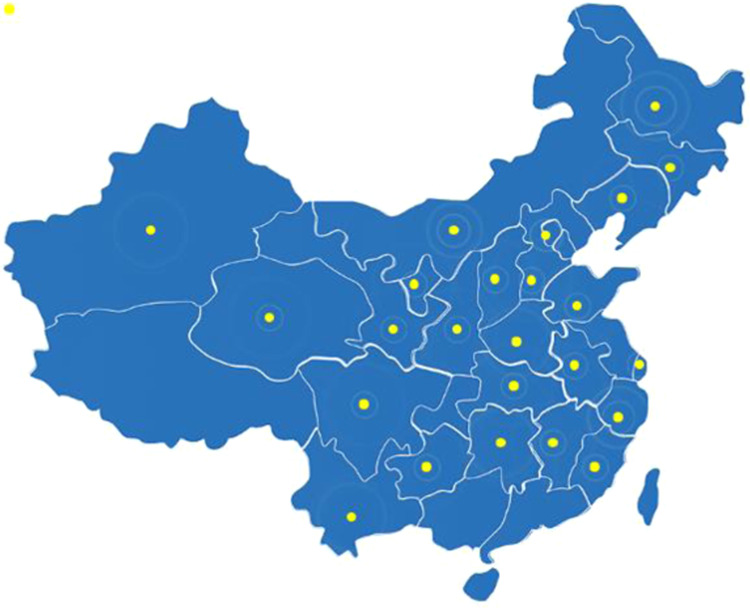
Branches of CARDPC. A map indicating where the branches of CARDPC can be found.

At the end of 2019, there were branches of CARDPC spread respectively in 29 out of 34 provinces or municipalities, the remaining 5 are planned (Fig. 1). The organization holds annual academic conferences, and set up the ‘Song Qingling Excellent Physicians in Primary Care Award’ for primary care physicians specifically, as well as a series of efforts to improve the ability of primary care physicians to manage respiratory diseases. This include the ‘Action Now Program, registration of COPD in national health system level’ [https://www.cardpc.org/pccm/guides/]. In the past few years, CARDPC has set up a series of respiratory diseases management training [https://www.cardpc.org/pccm/guides/] involving more than 200 sessions annually in every city in China.

It also launched the ‘Prevention and Treatment System and Capacity Building Project of Respiratory Diseases in Primary Care’ [https://www.cardpc.org/pccm/guides/], which has comprehensively improved the capacity of diagnosis and treatment of respiratory diseases in primary medical facilities. This has focused on three aspects: accessibility of equipment and drug; teaching of essential knowledge of chronic respiratory diseases management, and establishing medical consortia between primary medical facilities and higher-level medical institutions.

Furthermore, under the supervision of CARDPC, the first guidelines for COPD^4^, influenza^5^ and community acquired pneumonia (CAP)^6^ management in primary care have been released. Medications for these common respiratory diseases have been introduced to primary care facilities in some areas with the help of CARDPC, and competitions and lectures on respiratory diseases for primary care physicians are held every year to help improve physicians’ knowledge and skills.

As a member of the International Primary Care Respiratory Group (IPCRG), CARDPC actively participates in primary care research work and has international collaborations on respiratory diseases. CARDPC also pays great attention to primary care professionals’ research ability, and jointly with IPCRG set up the ‘Scientific Research and Training Camp for Chinese Primary Care Physicians’ [https://www.cardpc.org/article/detail/136] to improve the research ability of primary care professionals.

Altogether, this work has laid a foundation for primary care to respond to the COVID-19 emergency.

## Using social media to support the COVID-19 response

The leading academic organization of county hospitals and township health centers is the Alliance of County Hospitals in China (ACHC) [http://news.eastday.com/eastday/13news/auto/news/china/20180520/u7ai7728915.html]. To strengthen the prevention and control of COVID-19 in rural areas, CARDPC cooperated with ACHC to improve their ability to respond to COVID-19 at the county level.

**Fig. 2:**
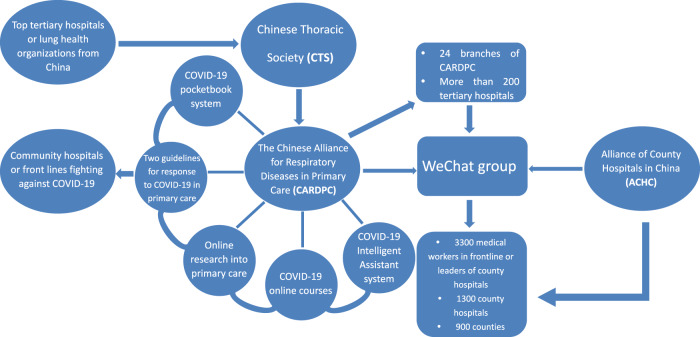
Actions undertaken by CARDPC and its interactions with other organizations.

WeChat, a social media application that is prominent in China, has played an important role in the communication and professional support during the pandemic. A WeChat social media group to provide remote guidance work of COVID-19 for 24 provinces or cities where suffered from serious outbreak was developed rapidly at the very beginning of the pandemic in China. In total, more than 200 hospital specialists in relevant fields were invited to the WeChat group, covering more than 3,300 medical professionals working in the frontline in 1300 county hospitals from 900 counties. Through this WeChat group, high-quality support and guidance from specialist was delivered quickly and efficiently to rural areas, which improved the capabilities of county hospitals and alleviated the work pressure on other facilities. By the end of February, a total of 647 clinical staff from 500 hospitals across the country participated in the interaction within the social media group; 1237 topics were discussed, including the criteria for discharge, how to collect specimens from patients with fever, how to support diagnosed children, how to manage chronic respiratory disease. 80.8% (999/1237) of these topics were related to capabilities of prevention and control of COVID-19 and 96.1% (960/999) of these questions were answered by the specialists instantly. Questions and answers discussed within the group were daily posted on the official WeChat account of CARDPC to benefit a wider range of healthcare workers [https://mp.weixin.qq.com/mp/homepage?__biz=MzUyMTk3NjQ0OQ==&hid=10&sn=fb3f660b5db34c84d23e18cc89cdaf2b&scene=1&devicetype=android-29&version=27000e37&lang=zh_CN&nettype=cmnet&ascene=59&session_us=gh_225939cc7806&wx_header=1]. The actions undertaken by CARDPC and its interactions with other organizations were outlined in Fig. 2.

## Publishing community prevention and control guidance

To strengthen and standardize the prevention and control of COVID-19 at the primary care level, CARDPC invited experts from respiratory medicine, general practice medicine, public health, epidemiology and other relevant fields to jointly draft the ‘Expert recommendations for the prevention and control of COVID-19 (First Edition)’ [https://www.ipcrg.org/resources/search-resources/expert-recommendations-for-the-prevention-and-control-of-covid-19]. The guide provides detailed guidance, including prevention and control regulations for general and special populations and the application of information technology in public health education. The guidance played an important role in prevention and control of COVID-19 at a community level across the country.

In addition, 10 online meetings for primary care physicians were held by CARDPC, experts in various disciplines were invited to interpret the guidelines and answer questions to facilitate its implementation. The content of online meetings were designed carefully, including basic knowledge of COVID-19, essential points of prevention and control, how to achieve a close connection between hospitals and primary care institutions, the management of elderly patients and those with chronic diseases under this pandemic, and so on. A total of 40,000 people attended these meetings, which had a very positive impact on the work of primary care medical facilities during the pandemic. The guidelines were published in *the Chinese Journal of General Practice* and have subsequently been made available in English by IPCRG [https://www.ipcrg.org/resources/search-resources/expert-recommendations-for-the-prevention-and-control-of-covid-19].

In a second publication, experts from CARDPC compiled the ‘Handbook on COVID-19 infection prevention and control in communities’ [https://www.ipcrg.org/resources/search-resources/handbook-on-covid-19-infection-prevention-and-control-in-communities] which contains 167 frequently asked questions and integrated common characteristics of communities. The manual provides detailed guidance on how to disinfect patients when transporting them, health education for the general population, psychological care for both patients and health care workers and interpretation of relevant national policies, i.e. Principles of Further Strengthening the Prevention and Control of Communities (Villages) during COVID-19 [http://www.beijing.gov.cn/zhengce/zhengcefagui/202002/t20200210_1627106.html?ivk_sa=1023197a]. The manual was released to primary medical staff, this has also been made available in English [https://www.ipcrg.org/resources/search-resources/handbook-on-covid-19-infection-prevention-and-control-in-communities].

Finally, to allow primary care professionals to view and search for COVID-19-related resources more easily and quickly, the “COVID-19 Pocket Book” system [https://mp.weixin.qq.com/mp/homepage?__biz=MzUyMTk3NjQ0OQ==&hid=10&sn=fb3f660b5db34c84d23e18cc89cdaf2b&scene=1&devicetype=android-29&version=27000c50&lang=zh_CN&nettype=WIFI&ascene=7&session_us=gh_225939cc7806&wx_header=1] was developed. Electronic document management system and the Q & A format were used to integrate the questions and answers raised in the WeChat group of the Alliance of County Hospitals, training materials for primary care specialists, and relevant guidelines and policies, which were all distributed on the WeChat account platform of CARDPC in different categories, and complemented with live broadcast and WeChat groups to form professional, real-time and flexible comprehensive online professional support.

## Online teaching courses for primary medical facilities

To answer the practical questions of primary health care staff and inform them how to protect themselves, CARDPC designed a series of COVID-19 teaching courses for primary health care institutions and themes included: general knowledge of COVID-19, screening, management, protective measures, diagnosis, treatment, prevention and control, and information synthesis. The courses have been published in stages on the WeChat account of CARDPC since February 2020, and 11 have been released to date.

In addition, CARDPC has launched 14 live broadcasts since February, 2020. The specific contents have included: important points of prevention and control of COVID-19, patient education, community management norms and principles of patients with hypertension, diabetes mellitus, coronary heart disease, cerebrovascular disease and COPD during the pandemic, as well as principles of health management for special groups: such as older people, children, patients with chronic diseases, pregnant women and those who have just given birth.

## Online auxiliary diagnosis support using artificial intelligence

CARDPC used artificial intelligence to develop the ‘COVID-19 Intelligent Assistant System’ [https://zgjchxjbfzlm.qiyukf.com/client?k=233462e82667c517ec95edc726928192&wp=1], which collates the guidelines of diagnosis, treatment, and prevention of COVID-19. It can accurately locate and provide the most professional information required by primary care physicians by raising questions online. The system also provides online auxiliary support for diagnosis, which can automatically reply or jump to the right pages by typing keywords into the system.

## Online research into primary care medical facilities

To fully understand the difficulties encountered by primary care medical facilities and their pressing needs, and to put forward practical measures to help primary care physicians to do a good job in prevention and control of the pandemic at the community level, CARDPC designed and implemented an online questionnaire to provide urgent help and support to primary care teams. The questionnaire was sent to 114 facilities by e-mail on 6^th^ February, 2020 [https://www.cardpc.org/]. By the end of April, a total of 91 questionnaires had been received from 18 cities. The feedback highlighted that primary care faced many difficulties such as shortage of protective clothing and other prevention materials, intensive and heavy burden of work of medical staff, insufficient manpower, and lack of guidance on prevention and control work. In response, CARDPC contacted businesses to provide or donate prevention and control materials to primary medical facilities in a very short timeframe, and developed the pocket book systems and training courses, as described above.

## Lessons learnt and future goals

In the process of fighting against COVID-19, CARDPC has done a lot of effective work, which is of great help to the prevention and control of the pandemic in communities’ level. However, we have also learned some lessons from the past months. Firstly, most of the specialists of CARDPC were from top hospitals or lung health organizations, even though few of them worked in primary care settings, we can’t learn the real situation and get a full picture of primary medical facilities in the first time. Secondly, as a link between primary care medical facilities and tertiary care, CARDPC can assemble high-quality medical resources and provide support from expert teams in a very short time, but the alliance cannot completely replace the work of primary care physicians, improving their ability is essential. Thirdly, although primary health care is responsible for the management of common diseases, it must have the ability to deal with public health emergencies and make preparations in advance. This health emergency also showed us the weaknesses that should be improved in the management of infectious respiratory diseases at primary care level in China in the future, such as how to quickly identify infectious respiratory diseases, how to manage chronic respiratory diseases, e.g. asthma and COPD, in COVID-19 context, and how to distinguish them from COVID-19.

Past experience of SARS has been used to guide clinical practice^7^, but in COVID-19, primary health care facilities in China have played an unprecedented role. Primary care also plays an important role in health systems to enable universal access to care, and the ability of physicians in primary care will directly affect the overall level of the management of both infectious and chronic respiratory diseases in China. Strengthening the capability to manage COVID-19 and those most at risk in primary care has begun to build strength in primary care respiratory disease management, which is the foundation to improve the overall quality of prevention and control of respiratory diseases in the whole country. Primary medical institutions and physicians are also the main force to achieve the goal of “healthy China 2030” [http://www.gov.cn/xinwen/2019-07/15/content_5409694.htm]. In the future, more physicians engaged in primary care would be recruited to CARDPC and we will continue to work hard to improve and prioritize the management of respiratory diseases in primary care.
